# Optical Properties and Sensing Performance of Au/SiO_2_ Triangles Arrays on Reflection Au Layer

**DOI:** 10.1186/s11671-018-2755-3

**Published:** 2018-10-24

**Authors:** Xianchao Liu, Jun Wang, Jun Gou, Chunhui Ji, Guanhao Cui

**Affiliations:** School of Optoelectronic Science and Engineering, University of Electronic Science and Technology of China, Chengdu, 610054 People’s Republic of China

**Keywords:** Localized surface plasmon, Triangle arrays, Absorber, Sensing, Figure of merit

## Abstract

In order to enhance the refractive index sensing performance of simple particle arrays, a structure, consisting of Au/SiO_2_ triangle arrays layers and reflection Au substrate, with increasing size and lengthening tips of triangles, is studied. The triangle arrays are modeled after an experimentally realizable “imprint” of microsphere lithography. Numerical calculation was carried out to study its optical properties and spectral sensitivity. The calculation results show that a large local enhancement of electric field (61 times) and simultaneously high absorption is due to combination of the resonance absorption of Au triangle disks, plasmonic couplings between the Au triangle disks and the Au film, and the high-density packing of triangle disks. The absorption peaks were not detuned when the gap between neighboring tips of the triangles varied from 10 to 50 nm. When the thickness of SiO_2_ layer increased from 10 to 50 nm, the absorption peak shifted to longer wavelengths and the amplitude rises quickly signaling the dominance of the gap mode resonance between the two Au layers. As the thickness of the top Au layer varies from 10 to 50 nm, the absorption peak is also red shifted and the peak amplitude increases. The full width at half maximum of the peaks for high absorption (> 90%) is about 5 nm. When fixing the gap, the thicknesses of Au/SiO_2_ triangle layer, and increasing the surrounding refractive index from 1.33 to 1.36, the absorption peaks shifted quickly, with a refractive index sensitivity and figure of merit as high as 660 nm per refractive index unit and 132, respectively. Such arrays can be easily fabricated by using microsphere array as projection masks and find application in refractive index monitoring of liquid and identification of gas and liquid phases.

## Highlights

1. The uniform MIM triangles structure with prolonged and sharp tips promises enhanced local electromagnetic field and extremely narrow band absorption.
2. The dense arrangement of the MIM triangles structure promises the high absorption.
3. The extremely narrow FWHM of absorption peak contributes to the high-performance refractive index sensing of the structure.

## Background

Localized surface plasmon resonances (LSPRs) carried by metallic nanoparticle and nanostructure arrays can capture light into themselves [1–3]. Especially, when they are small or with sharp edges, extreme high local electromagnetic field will occur among nanoscale spatial regions. The phenomenon attracts researchers’ extensive attention. Various structures, with patterned monolayer metal films, or metal/dielectric/metal multilayers, showing excellent performance of optics or electronics, have been suggested as plasmon sensor [4], broadband absorber [5, 6], surface enhanced Raman scatterer (SERS) [7, 8], transparent conducting metal [9, 10], and polarization converter [11]. However, commonly used lithography methods [12], like electron beam lithography, focused ion beam etching, and double beam interference lithography, are not suitable for fabricating large-area super-resolution pattern arrays, especially for patterns with sharp tips for high-performance field enhancement and sensing application, due to their high cost, low output, low lithography resolution, or poor flexibility. Thanks to the micro/nanosphere-assisted lithography, large-area triangular, crescent-shaped, hexagonal star-like pattern arrays with extreme sharp corners can be easily obtained [13–19], which can easily find application in sensing fields [16–19]. Of course, some similar patterns, like polygonal nanoprisms and metallic nanospheres, can also be obtained by a chemical synthesis method [20, 21] and it is also low cost. But the sharp degree of obtained prisms is not as good as that of the patterns obtained by sphere-assisted lithography. Microsphere lithography shows various advantages.

The refractive index sensing performance is evaluated by full width at half maximum (FWHM) of a resonance, the refractive index sensitivities (RIS), and figure of merit (FOM: RIS/FWHM). The usual method is to design a structure with small resonance linewidths and high RIS, resulting in big FOMs. Recently, Giuseppe Strangi’s team successfully fabricated a hyperbolic metamaterial biosensor, which consists of alternating films of thin Al_2_O_3_ and gold layers and achieves RIS of 30,000 nm per refractive index unit (RIU) [22]. The Bin Ren group has engineered the resonance linewidths by modulating the material, size, morphology of nanostructure, and ultranarrow FWHM of resonances down to 3 nm has been obtained in experiments [23]. The performance of sensors in Ref. [22, 23] is outstanding but disadvantages are low absorption of narrow resonance and complicated fabrication craft. The sensing performance of triangular surface patterns is usually higher than other kinds of the same structure with different morphology patterns due to triangles’ sharp tips. In the past, researchers mainly chose spheres with a diameter about 500 nm or smaller to fabricate triangular pattern arrays as small metallic particles usually provide high local electromagnetic field [18, 19]. The extinction or absorption of these small metallic particles lies in visible light and near ultraviolet. As for the existing size deviation of spheres and the actual gap difference between arbitrary neighboring spheres, the size of each fabricated triangle is with big deviation, which will result in widening of the FWHM of extinction/ absorption spectrum [18, 19]. Meanwhile, the RIS and FOM are generally small than 500 nm/RIU and 50, respectively, which limit its application in high-precision detection of solution index.

In addition, research of various recent literature suggests that compared with methods of controlling electromagnetic wave in monolayer metal pattern devices, there are more strategies to capture electromagnetic wave for MIM structure array devices [24–28], such as light coupling to a Fabry-Perot cavity, diffractive coupling in periodic arrays (Fano interference), and coupling to propagating surface plasmons. Monolayer metal disk array devices exhibit disadvantages in sensing performance.

To overcome the problems listed above, we suggest utilizing a bigger sphere to improve the size uniformity. A bigger sphere also means longer physical cross section of triangles, which will enhance the sensing performance of triangles. Our suggested structure contains three layers: the top Au layer and middle SiO_2_ layers are overlapping triangular patterns, while the bottom layer is Au reflection film, which can be fabricated by utilizing a microsphere array mask. We investigate the resonance absorption mechanism of the proposed structure, the gap size between adjacent tips of triangular patterns, and the thicknesses of SiO_2_ layer and Au layer influence on the position and amplitude of absorption peak. Lastly, optimization structure parameters are chosen, and we calculate the sensing properties of the structure. The obtained results of RIS and FOM are 660 nm/RIU and FOM 132, respectively, which are much better than former reports.

## Methods

**Fig. 1:**
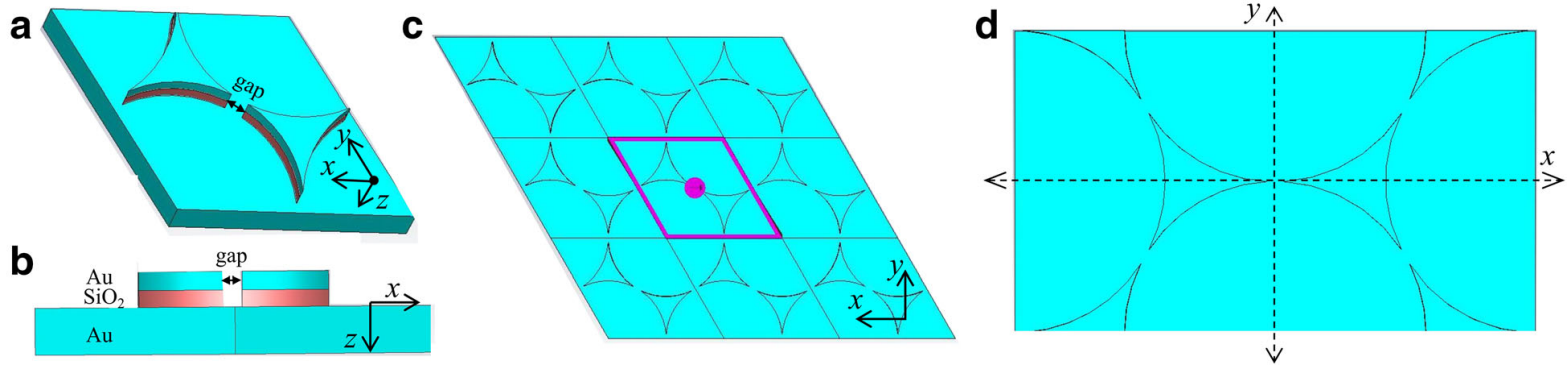
Schematic of MIM structure sensor. **a** Perspective view. **b** Cross-sectional view. **c**, **d** Top view

CST Microwave studio software is utilized to calculate electromagnetic field distribution and absorption of the three-layer structure. The schematic of metal/dielectric/metal (MIM) structure is shown in Fig. 1, which can be realized by micro/nanosphere array-assisted lithography [13, 29, 30]. Figure 1a–c shows perspective view, cross-sectional view, and top-view images, respectively, of the MIM structure array sensor and the structure model with boundary condition of unit cell in *xoy* plane (clearly seen in Fig. 1c), and open boundary conditions imposed at the model domain edge along the *z*-axis is set to calculate S parameters utilizing frequency domain solvers. Figure 1d is a top view of structure array, and periodic boundary in *xoy* plane and open boundary conditions at the model edge along the *z*-axis are set to calculate electromagnetic field distribution utilizing time domain solvers. Perfect matching layers are imposed outside of the open boundary along the *z*-axis. Adaptive mesh refinement is applied in all calculation and the solving accuracy is − 60 dB. The plane wave, with incident direction along the *z*-axis and polarization direction along the *x*-axis (for calculation of electromagnetic field), is set, whose amplitude is 1 V/M. The optical constant of materials is taken from Ref. [31]. During the simulation, the center-to-center spacing of adjacent triangles is fixed at 900 nm, while the gap between the tips of the adjacent triangles, the thickness of the middle dielectric layer and that of the top metallic layer, is adjusted. Absorption spectra and spectral shifts are obtained. By varying the environment refractive index, the sensitivity of spectral to external material changes is obtained. The calculation results and analysis are as follows.

## Results and Discussion

### Optical Properties

**Fig. 2:**
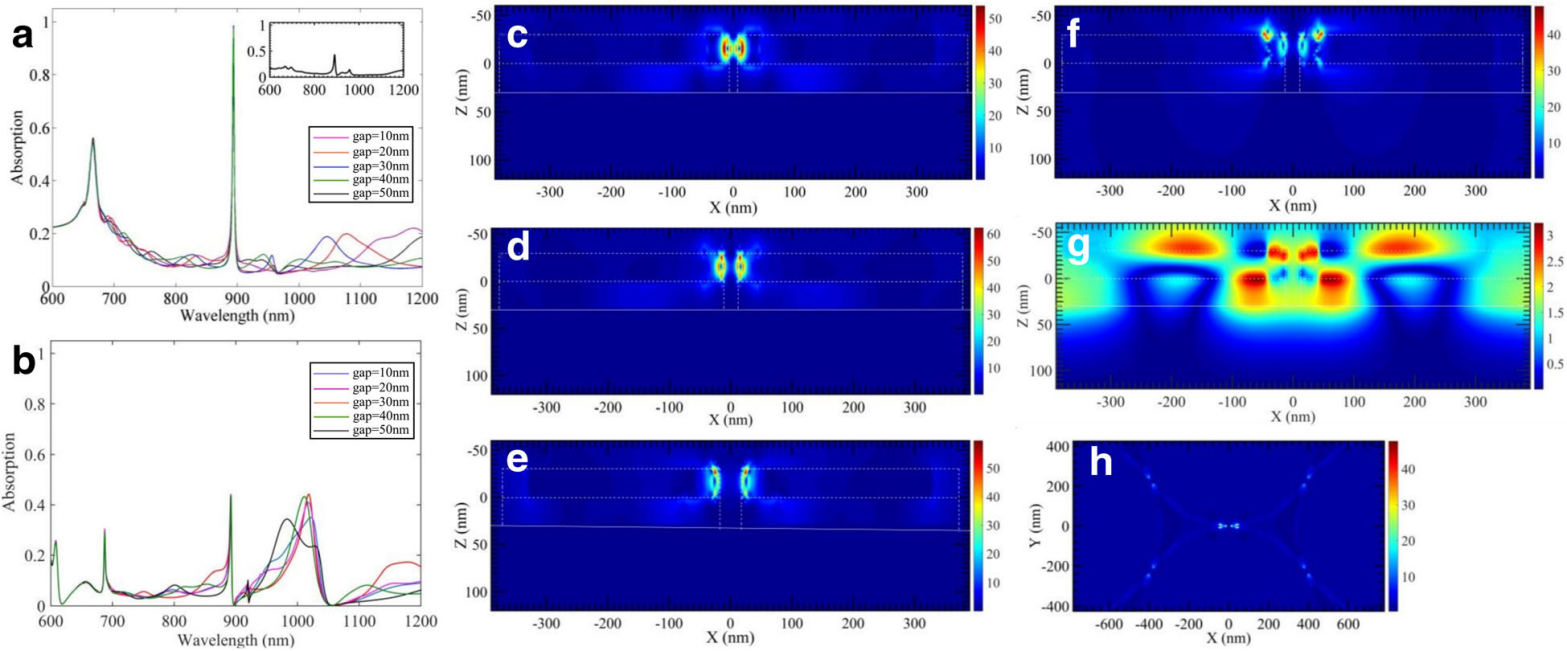
Absorption spectrum varies with the gap sizes between adjacent tips of triangles increasing of MIM structure array (**a**) and MII structure array (**b**). The inset at the top-right corner of **a** is absorption spectrum of isolated MIM structure. **c**–**e** Electric field *|E|* distribution of *xoz* plane (*y* = 0 nm) of MIM structure array models with gap sizes of 20 nm, 30 nm, 50 nm, respectively. **f** *|E|* distribution of *xoz* plane (*y* = 0 nm) of MII structure array model with gap size 30 nm. **g** *|H|* distribution of *xoz* plane (*y* = 0 nm) of MIM structure array model with gap size of 30 nm. **h** *|E|* distribution of *xoy* plane (*z* = − 30 nm) of MIM structure array model with gap size of 30 nm

The structure parameters of the MIM structure are systematically varied. First, the top Au and middle dielectric layers are set as 30 nm and 30 nm, respectively. The bottom Au film is 100 nm, which is thick enough to reflect all the light. The transmission *T* is nearly 0 [24]. The absorption *A* can be obtained using 1-R (R: reflectivity by the model). The refractive index of the environment is 1.34. In order to know how the gap between the adjacent tips of neighboring triangles affects absorption peak, we study the relation between the absorption spectrum and gap between neighboring tips first. The results are presented in Fig. 2. Figure 2a shows the absorption spectra of the MIM structure array with the gap sizes 10 nm, 20 nm, 30 nm, 40 nm, and 50 nm. From the spectra, we see the tip gap (varying among 10~50 nm) does not affect the position and amplitude of the main peaks (at ~ 900 nm), suggestive of its association to another resonance modes. Following the MIM structure array with a 30-nm gap size, a MIM structure array model with halving triangle in each unit is built for further analysis. The smallest gap size between adjacent triangles of the model with a sparse triangle arrangement is bigger than 500 nm, where no interaction exists between them. We calculate the S parameter of the model, whose absorption spectrum is the inset of Fig. 2a. The position of main peak is nearly the same with that of MIM structure array with small gap size (varying among 10~50 nm), while the absorption of the peak reduces a lot. Thus, it can be concluded that the formation of the main peak is mainly related to isolated MIM unit. To further confirm the forming reason of the main peak, models, keeping the gap size (varying among 10~50 nm) and replacing the bottom Au film with SiO_2_ film, are built. The absorption of the changed models (metal/dielectric/dielectric, MII) is shown in Fig. 2b. The peaks near 900 nm in Fig. 2a, b are with nearly the same position and FWHM, but the amplitude of the latter is far less than that of the former. It can be concluded that the forming reason of the main peaks in MIM structure array is attributed to the patterned top and middle layers. Meanwhile, the reflection Au substrate of MIM structure plays an important role in enhancing the absorption. For the MII structure, there exist LSPRs and surface lattice resonance (SLR) [28]. The peak position of SLR is at ~ 1000 nm, which is the result of LSP mode of one Au disk with coherent diffraction coupling compared to other Au disks. As the thickness of SiO_2_ is too thin, SLR does not observed in MIM structures. As the polarization influences the absorption spectra of MIM structure arrays slightly [32, 33], we do not discuss it here.

To analyze the detail, a periodic model, with top view as shown in Fig. 1d, illuminated by a linear polarization light source (wavelength of 893.8 nm that is the position of the main peak), is built. The electric field *|E|* is given in Fig. 2c–g. Figure 2c–e is electric field distribution of *xoz* plane (*y* = 0 nm), with gap size of 20 nm, 30 nm, and 50 nm, respectively. The maximum *|E|* occurs among the gap of adjacent Au triangles for condition of gap size of 10 nm, and at the tips of Au triangles for bigger gap sizes. The maximum value varies from 54 to 61, which is a slight fluctuation. However, the electric field among SiO_2_ layer is extremely low. It is the same situation with that of the MII structure array, with a gap size of 30 nm, shown in Fig. 1f. The maximum field occurs also at the tips of Au triangles, about 48, which is a little smaller than that of the MIM structure array model with same gap sizes. The electric field of the SiO_2_ layer is close to zero, while magnetic field *|H|* is enhanced, as shown in Fig. 2g. The *|H|* can be improved by adjusting thickness of spacer and Au triangles. Comparing with previous research on MIM structure absorbers [32, 34] and our finding, it can be concluded that although coupling may exist between adjacent Au triangles, small changing of this kind of triangles (with very long and sharp tips) will not result in movement of the main peak and reduction of the enhanced local field. The local enhancement of electric field (~ 48 times of incident field) at tips of isolated Au triangles is due to the tip size effect or lighting rod effect [33, 35], which results in ~ 42% absorption of the main peak of MII structure models. The large local electric field (> 54 times of incident field) and high absorption (> 90%) of the main peaks should be ascribed to the simultaneous lighting rod effect of Au triangle disks and the fundamental magnetic resonance mode among SiO_2_ spacer layers, which excite the MIM structure array responding to the incident light, resulting in ultranarrow FWHM of the main peaks with high absorption. The FWHM of its main absorption peaks is significantly smaller than that of the MIM structure with normal triangle disks [32], which benefits its sensing performance. The decrease of absorption of MIM with halving triangle in each unit is due to a low density of “hot spots” [36]. In addition, the reflect Au also provide extra opportunity for LSPR absorption among Au disks. Thus, the field enhancement of triangle MIM structure array is a little higher than that of monolayer triangle array on Si [37]. Lastly, the electric field of *xoy* plane (*z* = − 30 nm, the upper surface of top Au layer) of the MIM array model is given in Fig. 2h. Clear bright spots can be seen at all tips of the Au triangles. However, it can be observed that the spots lied in the center line, which is parallel to the *x*-axis (the polarized direction of illumination) of a vertex of a triangle and is brighter. The phenomenon is in accord with the results shown in Ref. [37, 38], which indicates that part of the main electric field contribution comes from the in-plane component parallel to the incoming light.

**Fig. 3:**
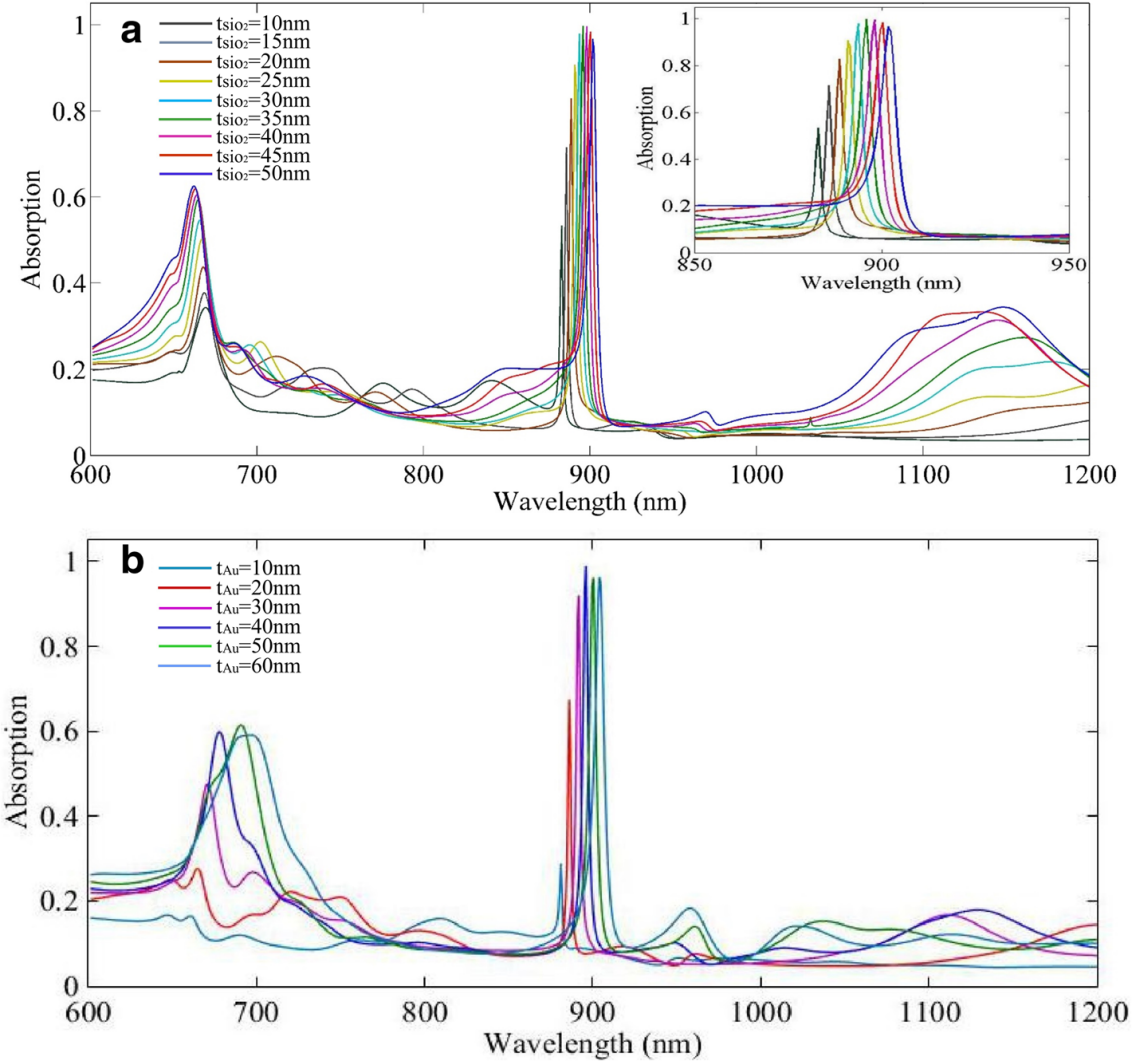
**a** Absorption spectrum varies with thickness of SiO_2_ layer increasing. **b** Absorption spectrum varies with thickness of top triangle Au array layer increasing

As the gap between neighboring triangles exists in the experiment, and precise controlling of gap size (accuracy ~ 15 nm, minimum mean gap value 10 nm) is possible by several methods [29, 30], we choose to have the gap size fixed at 30 nm in the following study. Then, the thicknesses of the middle SiO_2_ layer and top Au layers are varied, respectively. When the thickness of SiO_2_ layer increases, the position and amplitude of the absorption peaks change quickly, which is shown in Fig. 3a. When the SiO_2_ layer is thin, there just exist LSPR absorption and the absorption of peak at ~ 900 nm is low. With the increasing of thickness of SiO_2_ layer, red shift of peaks occurs and the absorption reaches 90%. The reason for the red shift of peaks is that when the thickness of the SiO_2_ layer increases, the effective refractive index surrounding the triangle arrays increases, which results in red shift of plasmon peaks. Meanwhile, magnetic resonance forms in the SiO_2_ layer. The electric resonance (from LSPRs) inside the Au triangles combining with magnetic resonance responds to incident light, resulting in extreme high absorption at ~ 900 nm. Furthermore, the sharp tips of triangles promise the narrow FWHM of peaks. For the thickness range of the SiO_2_ layer, 25~40 nm, the absorption is higher than 90%, but the FWHM of peak is a little smaller when the SiO_2_ thickness is 25 nm. It is because more intense coupling between electric and magnetic modes occurs. Thus, we choose 25 nm of SiO_2_ and continue to study the top Au layer effect on optical properties of the MIM structure sensor. The relationship is shown in Fig. 3b. The absorption is low when the thickness of Au triangles is 10 nm. When the thickness increases, the peak position is red shifted and the amplitude increases. When the thickness increases to 30 nm, the amplitude reaches 90%. With the continuing increase of thickness of the top Au layer, the absorption does not vary while the FWHM widens. The FWHM varies from 3.5 to 6 nm. It should be attributed to increasing ohmic loss with increasing thickness of the top Au film. We choose the top Au layer of 50 nm as an appropriate parameter for the MIM sensor, and the FWHM of the peak is 5 nm. The reason for the red shift is that when the thickness of Au triangles increases, the number of free electrons engaging in the collective shock increases and the delay effect of the electromagnetic field inclines; thus, the energy required for the equal resonance excitation is reduced [39]. As lots of free electrons engage in resonance, the amplitude rises and FWHM of peak is extremely narrow. The peak position is related to the sharpness and geometric dimensions of the triangles, and the number of free electrons accumulated at the tips of the triangles is large, the energy required for the resonance excitation is small, and the resonance wavelength is red shifted.

### Sensing Performance

**Fig. 4:**
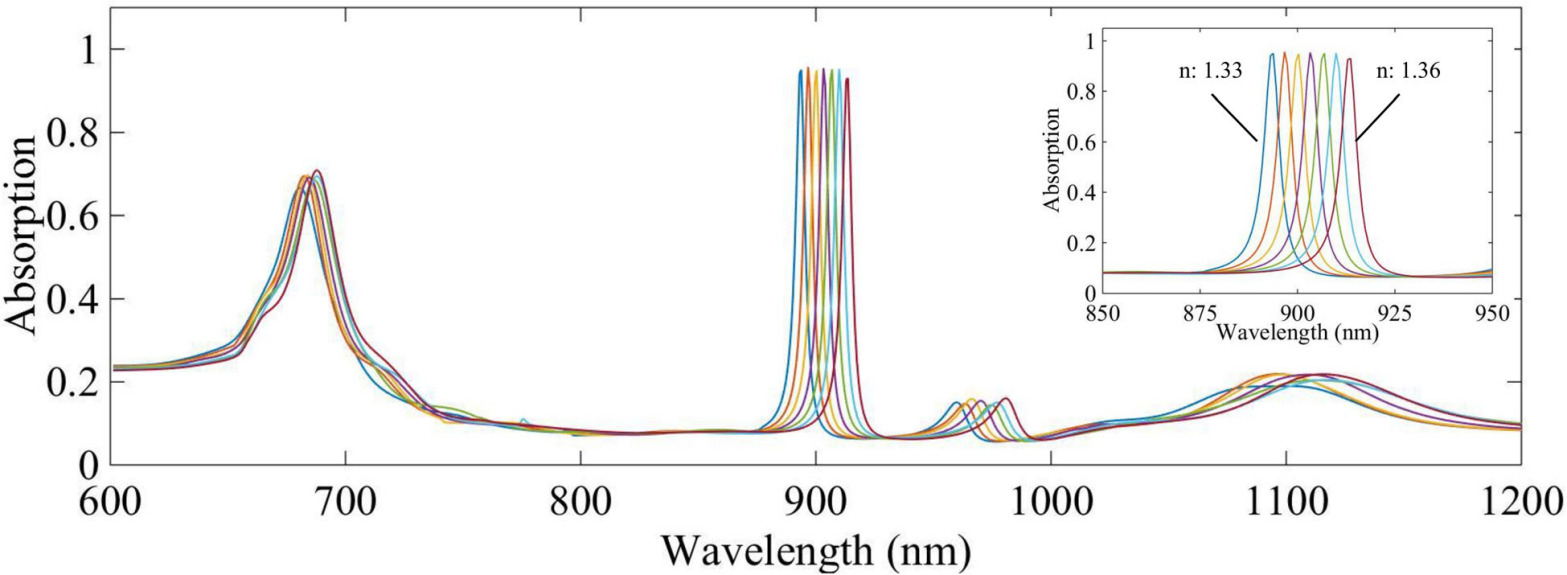
Absorption peak varies with environment refractive index (from 1.33 to 1.36) increasing

In the study above, we have arrived at optimized parameters of gap size between neighboring tips of triangle disk, thickness of SiO_2_ spacer, and top Au disk, which are 30 nm, 25 nm, and 50 nm, respectively. In this part, the already optimized parameters are fixed, and the absorption spectrum varying with environment refractive index is calculated and shown in Fig. 4. With the refractive index of environment increasing, quick red shift of extreme narrow, high absorption peaks can be seen. The FWHM for each peak is about 5 nm. We calculate the RIS and FOM, which are about 660 nm/RIU and 132, respectively. The optimization results of sensing properties by numerical study of the conventional patterns are excellent. Thanks to small size deviation of commercially available microspheres, mature microsphere self-assembly technology, and also the methods of precise control gap size [29, 30], the suggested MIM structure sensor can find practical application in detection of solution index and identification solutions.

## Conclusions

Numerical calculation is carried out to study optical properties and sensing performance of MIM structure sensor with patterned triangle unit. The enhanced local electric field and high absorption simultaneously is attributed to the strong lighting rod effect of Au triangle disks, plasmonic resonance coupling of electric resonance among Au triangle disks and magnetic resonance which dwelled in the SiO_2_ layer, and high-density arranged triangle MIM arrays. The interaction among adjacent triangle disks of our structure and parameter effect on absorption peak is negligible. The thicknesses of the SiO_2_ layer and top Au layer influence the position and amplitude of peaks, which are caused by adjusting electric dipoles and magnetic dipoles of MIM structure to match impedance, and the increasing of geometric dimensions of triangles when the thickness of SiO_2_/Au triangle layer increases. When the suggested structure matches its effective impedance well, the absorption is extremely high (> 90%). Due to the long tips of triangle Au arrays, the FWHM of peaks is very narrow, about 5 nm. The obtained RIS and FOM are about 660 nm/RIU and 132, respectively, for environment refractive index 1.33~ 1.36, which are excellent results compared with previous reports.

## Abbreviations

Al_2_O_3_: Aluminum oxide
FOM: Figure of merit
FWHM: Full width at half maximum
LSPR: Localized surface plasmon resonance
MII: Metal/dielectric/dielectric
MIM: Metal/dielectric/metal
RIS: Refractive index sensitivities
RIU: Refractive index unit
SiO_2_: Silicon dioxide
